# Supplementary material for: Effect of an antenatal diet and lifestyle intervention and maternal BMI on cord blood DNA methylation in infants of overweight and obese women: The LIMIT Randomised Controlled Trial
Source: PLoS One. 2022 Jun 24;17(6):e0269723. doi: 10.1371/journal.pone.0269723 (PMC9231808; doi:10.1371/journal.pone.0269723)
Supplement: S1 Table — (DOCX) [file pone.0269723.s001.docx]

Supplementary Material: S1 Table

Effect of an Antenatal Diet and Lifestyle Intervention and Maternal BMI on Cord Blood DNA Methylation in Infants of Overweight and Obese Women: the LIMIT Randomised Controlled Trial

**Table 1: Genes/loci reported in other studies to be differentially methylated in cord blood in association with maternal BMI/obesity**

| Gene/locus | Association | Reference |
| --- | --- | --- |
| *RXRA* | Higher methylation of 2 CpGs in the promoter associated with greater adiposity; associations also observed between *RXRA* methylation and maternal carbohydrate intake | [10] |
| *PPARGC1A* | Higher methylation in promoter region associated with higher maternal pre-pregnancy BMI | [19] |
| *ESM1*  *MS4A3* | Hypomethylation with increasing BMI | [16] |
| cg09243648  cg13403462 (*ACTL10; NECAB3)*  cg20594982 *(AGRN)*  cg18144647  cg14528056 *(GBAP1)*  cg01963618 *(LOC285768)*  cg05113927 *(UCN)*  cg05635274 *(PRSS21)* | 86 sites in total were determined to show evidence for a casual intrauterine effect of maternal BMI in the PACE consortium; these are the 8 for which there was strongest evidence. | [39] |
| unnamed (cg00526953)  *SUCLG2* (cg02321096)  *FAM129B* (cg03270036)  *KIF15;KIAA1143* (cg17546649)  *STAB2* (cg23131355) | 28 CpG sites with FDR adjusted p < 0.05 for association between maternal pre-pregnancy obesity and offspring cord blood methylation in the ALSPAC cohort.  Top 5 sites are shown. | [38] |
| *TAPBP* | CpG sites mapped to this gene were found to be differentially methylated for both male and female infants born to obese women in the NEST cohort. | [45] |
| *ZCCHC10* | Cord blood DNAm at one CpG site on this gene was significantly associated with maternal obesity | [40] |
| *unnamed (*cg12053563)  *unnamed (*cg12549355)  *FLJ41941 (*cg02975187)  *SFRS8* | Significant associations with maternal pre-pregnancy (*FLJ41941*),and with central obesity ( cg12053563, cg12549355, unnamed gene), as well as a “suggestive association” between BMI and *SFRS8.* | [41] |
| *PLAGL1 (imprinted gene DMR)*  *MEG3 (imprinted gene DMR)* | differential methylation associated with maternal obesity in cord blood for the DMRs related to these imprinted genes. | [42] |
| *MEST* | differential methylation i associated with morbid obesity compared to normal weight mothers; in addition there was differential methylation of this gene associated with GDM | [20] |
