# Supplementary material for: Effect of an antenatal diet and lifestyle intervention and maternal BMI on cord blood DNA methylation in infants of overweight and obese women: The LIMIT Randomised Controlled Trial
Source: PLoS One. 2022 Jun 24;17(6):e0269723. doi: 10.1371/journal.pone.0269723 (PMC9231808; doi:10.1371/journal.pone.0269723)
Supplement: S2 Table — (DOCX) [file pone.0269723.s002.docx]

Supplementary Material: S2 Tables

Effect of an Antenatal Diet and Lifestyle Intervention and Maternal BMI on Cord Blood DNA Methylation in Infants of Overweight and Obese Women: the LIMIT Randomised Controlled Trial

# Top 10 Tables (by p value) for Sensitivity Analysis Models

## Table 1 Unadjusted model

Not including maternal age, parity, smoking status, study centre and quintile of socioeconomic disadvantage, but still adjusted for batch and estimated cell type proportion.

| Intervention at Mean BMI | | | | | Intervention at BMI +5 | | | | |
| --- | --- | --- | --- | --- | --- | --- | --- | --- | --- |
| chr | Name | UCSC RefGene Name | logFC | adj.P.Val | chr | Name | UCSC RefGene Name | logFC | adj.P.Val |
| chr19 | cg03057840 |  | -0.10 (-0.13, -0.06) | 0.21 | chr19 | cg03057840 |  | -0.09 (-0.13, -0.05) | >0.99 |
| chr13 | cg20260570 | C13orf34;C13orf37 | -0.08 (-0.12, -0.05) | >0.99 | chr12 | cg09636302 | HAL | -0.12 (-0.17, -0.07) | >0.99 |
| chr21 | cg01233397 |  | 0.11 (0.06, 0.15) | >0.99 | chr21 | cg01233397 |  | 0.11 (0.06, 0.16) | >0.99 |
| chr11 | cg13932624 | TBRG1 | -0.06 (-0.08, -0.03) | >0.99 | chr11 | cg13932624 | TBRG1 | -0.06 (-0.09, -0.03) | >0.99 |
| chr13 | cg11421702 | C13orf37;C13orf34 | -0.08 (-0.12, -0.04) | >0.99 | chr13 | cg20260570 | C13orf34;C13orf37 | -0.08 (-0.12, -0.04) | >0.99 |
| chr12 | cg09636302 | HAL | -0.11 (-0.16, -0.06) | >0.99 | chr17 | cg04435975 | LOC404266;HOXB6 | 0.08 (0.04, 0.12) | >0.99 |
| chr17 | cg04435975 | LOC404266;HOXB6 | 0.08 (0.04, 0.11) | >0.99 | chr13 | cg11421702 | C13orf37;C13orf34 | -0.08 (-0.12, -0.04) | >0.99 |
| chr4 | cg14712262 | ZFYVE28 | -0.05 (-0.08, -0.03) | >0.99 | chr20 | cg11336672 | RBL1 | 0.14 (0.08, 0.21) | >0.99 |
| chr18 | cg17242353 |  | 0.13 (0.07, 0.20) | >0.99 | chr8 | cg19595092 | SLC39A14 | 0.10 (0.05, 0.14) | >0.99 |
| chr12 | cg11551902 | FOXM1;C12orf32 | -0.14 | >0.99 | chr3 | cg20559405 | PTPRG | -0.07 (-0.11, -0.04) | >0.99 |
| BMI In Standard Care | | | | | BMI in Lifestyle Advice | | | | |
| chr3 | cg25821785 | CACNA2D2 | -0.05 (-0.07, -0.03) | 0.14 | chr11 | cg07823293 | TBRG1 | 0.09 (0.05, 0.12) | 0.40 |
| chr18 | cg03776551 | ZNF396 | 0.05 (003, 0.07) | 0.48 | chr3 | cg11118235 | GNAI2 | 0.05 (0.03, 0.08) | 0.59 |
| chr7 | cg22005393 | DNAJC2 | -0.04 (-0.06, -0.03) | 0.48 | chr4 | cg12630714 |  | 0.07 (0.04, 0.10) | 0.59 |
| chr16 | cg06397424 | NDE1;KIAA0430 | -0.05 (-0.08, -0.03) | 0.48 | chr11 | cg13466901 | OMP;CAPN5 | -0.06 (-0.09, -0.04) | 0.75 |
| chr9 | cg01263574 | TMEM8C | 0.03 (0.02, 0.05) | 0.48 | chr2 | cg06695611 | ZNF385B;MIR1258 | -0.11 (-0.16, -0.06) | 0.75 |
| chr10 | cg18646207 | VAX1 | 0.08 (0.05, 0.12) | 0.48 | chr11 | cg01504836 | KDM4D;CWC15 | -0.08 (-0.12, -0.05) | 0.75 |
| chr6 | cg27244242 | LY6G5C | 0.03 (0.02, 0.04) | 0.48 | chr8 | cg16903025 | FBXO32 | -0.07 (-0.11, -0.04) | 0.78 |
| chr15 | cg26672604 |  | 0.06 (0.03, 0.08) | 0.48 | chr8 | cg17826753 | KCNV1 | 0.03 (0.02, 0.05) | 0.85 |
| chr2 | cg16639766 | HJURP | 0.06 (0.03, 0.09) | 0.48 | chr17 | cg08645827 | CCT6B | -0.05 (-0.07, -0.03) | 0.85 |
| chr10 | cg16310045 | TCF7L2 | -0.04 (-0.06, -0.02) | 0.51 | chr1 | cg08867825 | OLFM3 | 0.08 (0.04, 0.12) | 0.85 |

## Table 2 SWAN Normalised Data

Adjusted model as for main analysis (adjusted for maternal age, parity, smoking status, study centre, quintile of socioeconomic disadvantage, as well as batch and estimated cell type proportion).

| Intervention at Mean BMI | | | | | Intervention at BMI +5 | | | | |
| --- | --- | --- | --- | --- | --- | --- | --- | --- | --- |
| chr | Name | UCSC RefGene Name | logFC | adj.P.Val | chr | Name | UCSC RefGene Name | logFC | adj.P.Val |
| chr18 | cg17242353 |  | 0.16 (0.09, 0.23) | >0.99 | chr18 | cg17242353 |  | 0.16 (0.09, 0.23) | >0.99 |
| chr13 | cg20260570 | *C13orf34;C13orf37* | -0.11 (-0.16, -0.06) | >0.99 | chr13 | cg20260570 | *C13orf34;C13orf37* | -0.11 (-0.16, -0.06) | >0.99 |
| chr1 | cg04263115 | *CLSPN* | -0.12 (-0.17, -0.07) | >0.99 | chr1 | cg22559942 | *TIMM17A* | -0.07 (-0.11, -0.04) | >0.99 |
| chr21 | cg01233397 |  | 0.12 (0.07, 0.18) | >0.99 | chr21 | cg01233397 |  | 0.12 (0.07, 0.18) | >0.99 |
| chr4 | cg10202508 | *TMEM192* | -0.10 (-0.14, -0.05) | >0.99 | chr3 | cg20559405 | *PTPRG* | -0.08 (-0.12, -0.04) | >0.99 |
| chr12 | cg11551902 | *FOXM1;C12orf32* | -0.13 (-0.20, -0.07) | >0.99 | chr4 | cg10202508 | *TMEM192* | -0.09 (-0.14, -0.05) | >0.99 |
| chr3 | cg24837680 | *ZNF501* | -0.12 (-0.17, -0.06) | >0.99 | chr8 | cg20176371 | *MYOM2* | 0.08 (0.04, 0.12) | >0.99 |
| chr1 | cg22606205 | *PKP1* | -0.12 (-0.18, -0.06) | >0.99 | chr12 | cg09636302 | *HAL* | -0.12 (-0.18, -0.06) | >0.99 |
| chr3 | cg20559405 | *PTPRG* | -0.08 (-0.12, -0.04) | >0.99 | chr3 | cg24837680 | *ZNF501* | -0.11 (-0.17, -0.06) | >0.99 |
| chr8 | cg20176371 | *MYOM2* | 0.08 (0.04, 0.12) | >0.99 | chr17 | cg04435975 | *LOC404266;HOXB6* | 0.09 (0.05, 0.14) | >0.99 |
| BMI In Standard Care | | | | | BMI in Lifestyle Advice | | | | |
| chr7 | cg22005393 | *DNAJC2* | -0.06 (-0.09, -0.04) | 0.31 | chr1 | cg00492341 | *MMEL1* | -0.05 (-0.07, -0.03) | 0.82 |
| chr2 | cg00143045 | *RPRM* | -0.12 (-0.17, -0.07) | 0.31 | chr11 | cg12911952 | *SLC22A18AS* | -0.05 (-0.07, -0.03) | 0.82 |
| chr22 | cg25685359 |  | -0.07 (-0.09, -0.04) | 0.31 | chr11 | cg13466901 | *OMP;CAPN5* | -0.08 (-0.11, -0.04) | 0.82 |
| chr8 | cg23783862 | *CHMP4C* | 0.08 (0.04, 0.11) | 0.31 | chr1 | cg04868078 | *LHX9* | -0.12 (-0.17, -0.07) | 0.82 |
| chr2 | cg05223061 | *NGEF* | 0.10 (0.06, 0.15) | 0.31 | chr1 | cg08867825 | *OLFM3* | 0.12 (0.07, 0.17) | 0.89 |
| chr3 | cg25821785 | *CACNA2D2* | -0.06 (-0.09, -0.03) | 0.31 | chr14 | cg23428143 | *OTX2OS1* | -0.06 (-0.08, -0.03) | >0.99 |
| chr14 | cg07031872 | *SLC24A4* | -0.04 (-0.06, -0.02) | 0.31 | chr6 | cg15226808 |  | -0.05 (-0.07, -0.03) | >0.99 |
| chr10 | cg16310045 | *TCF7L2* | -0.06 (-0.08, -0.03) | 0.31 | chr13 | cg15244467 | *MYO16* | -0.05 (-0.07, -0.02) | >0.99 |
| chr3 | cg01919208 | *LAMB2* | -0.07 (-0.09, -0.04) | 0.31 | chr4 | cg23991169 | *PCGF3* | -0.04 (-0.05, -0.02) | >0.99 |
| chr14 | cg18696159 |  | 0.08 (0.04, 0.11) | 0.31 | chr2 | cg06695611 | *ZNF385B;MIR1258* | -0.10 (-0.14, -0.05) | >0.99 |

## Table 3 BMIQ Normalised Data

Adjusted model as for main analysis (adjusted for maternal age, parity, smoking status, study centre, quintile of socioeconomic disadvantage, as well as batch and estimated cell type proportion).

| Intervention at Mean BMI | | | | | Intervention at BMI +5 | | | | |
| --- | --- | --- | --- | --- | --- | --- | --- | --- | --- |
| chr | Name | UCSC RefGene Name | logFC | adj.P.Val | chr | Name | UCSC RefGene Name | logFC | adj.P.Val |
| chr13 | cg20260570 | C13orf34;C13orf37 | -0.16 (-0.2, -0.09) | 0.99 | chr13 | cg20260570 | C13orf34;C13orf37 | -0.16 (-0.22, -0.09) | >0.99 |
| chr18 | cg17242353 |  | 0.13 (0.07, 0.18) | 0.99 | chr11 | cg13932624 | TBRG1 | -0.13 (-0.18, -0.07) | >0.99 |
| chr11 | cg13932624 | TBRG1 | -0.12 (-0.18, -0.07) | 0.99 | chr8 | cg20176371 | MYOM2 | 0.15 (0.09, 0.22) | >0.99 |
| chr8 | cg20176371 | MYOM2 | 0.15 (0.08, 0.22) | 0.99 | chr22 | cg20950843 | CYB5R3 | 0.14 (0.08, 0.20) | >0.99 |
| chr1 | cg04263115 | CLSPN | -0.13 (-0.19, -0.07) | 0.99 | chr22 | cg09783309 | DNAL4 | -0.11 (-0.17, -0.06) | >0.99 |
| chr21 | cg01233397 |  | 0.20 (0.11, 0.29) | 0.99 | chr18 | cg17242353 |  | 0.12 (0.07. 0.18) | >0.99 |
| chr14 | cg23470598 | KLC1 | 0.14 (0.08, 0.21) | 0.99 | chr17 | cg04435975 | LOC404266;HOXB6 | 0.10 (0.06, 0.15) | >0.99 |
| chr22 | cg20950843 | CYB5R3 | 0.13 (0.07, 0.19) | 0.99 | chr21 | cg01233397 |  | 0.20 (0.11, 0.29) | >0.99 |
| chr4 | cg10202508 | TMEM192 | -0.10 (-0.14, -0.05) | 0.99 | chr3 | cg20559405 | PTPRG | -0.10 (-0.15, -0.05) | >0.99 |
| chr6 | cg22509399 | MSH5 | -0.16 (-0.23, -0.08) | 0.99 | chr8 | cg19595092 | SLC39A14 | 0.16 (0.08, 0.23) | >0.99 |
| BMI In Standard Care | | | | | BMI in Lifestyle Advice | | | | |
| **chr5** | **cg24630195** | **IRX2** | **-0.65 (-0.86, -0.44)** | **<0.001** | chr7 | cg05522288 | NAA38 | -0.45 (-0.63, -0.26) | 0.47 |
| **chr12** | **cg06103657** | **PKP2** | **-0.70 (-0.92, -0.47)** | **<0.001** | chr16 | cg27347003 | ATP2A1 | -0.51 (-0.73, -0.30) | 0.47 |
| **chr2** | **cg08280341** |  | **-0.65 (-0.87, -0.43)** | **<0.001** | chr12 | cg02727674 | FGD6;VEZT | -0.45 (-0.64, -0.26) | 0.47 |
| **chr5** | **cg17394978** | **IRF1** | **-0.63 (-0.85, -0.41)** | **<0.001** | chr1 | cg08867825 | OLFM3 | 0.15 (0.08, 0.21) | 0.47 |
| **chr19** | **cg04772683** | **KLK10** | **-0.17 (-0.23, -0.11)** | **<0.001** | chr4 | cg12630714 |  | 0.05 (0.03, 0.07) | 0.47 |
| **chr1** | **cg06353611** | **SCNM1;LYSMD1** | **-0.20 (-0.27, -0.13)** | **<0.001** | chr1 | cg00492341 | MMEL1 | -0.07 (-0.09, -0.04) | 0.47 |
| chr9 | cg13977604 | ALG2 | 0.11 (0.07, 0.15) | 0.05 | chr11 | cg07823293 | TBRG1 | 0.07 (0.04, 0.11) | 0.47 |
| chr2 | cg05223061 | NGEF | 0.13 (0.08, 0.19) | 0.07 | chr1 | cg04868078 | LHX9 | -0.12 (-0.18, -0.07) | 0.47 |
| chr7 | cg22005393 | DNAJC2 | -0.10 (-0.13, -0.06) | 0.07 | chr6 | cg15226808 |  | -0.07 (-0.11, -0.04) | 0.97 |
| chr14 | cg13170179 | GALNTL1 | -0.06 (-0.09, -0.04) | 0.09 | chr14 | cg23428143 | OTX2OS1 | -0.06 (-0.09, -0.03) | 0.97 |

## Table 4. ComBat-processed data

Model adjusted for confounders (maternal age, parity, smoking status, study centre, quintile of socioeconomic disadvantage) and estimated cell type proportions. ComBat procedure was implemented in supervised form (specifying Intervention, BMI and their interaction as target effects).

| Intervention at Mean BMI | | | | | Intervention at BMI +5 | | | | |
| --- | --- | --- | --- | --- | --- | --- | --- | --- | --- |
| chr | Name | UCSC RefGene Name | logFC | adj.P.Val | chr | Name | UCSC RefGene Name | logFC | adj.P.Val |
| **chr19** | **cg03057840** |  | **-0.09 (-0.13, -0.06)** | **0.03** | chr19 | cg03057840 |  | -0.09 (-0.12, -0.06) | 0.19 |
| chr13 | cg20260570 | C13orf34;C13orf37 | -0.08 (-0.11, -0.05) | 0.18 | chr12 | cg09636302 | HAL | -0.11 (-0.16, -0.07) | 0.20 |
| chr18 | cg17242353 |  | 0.14 (0.08, 0.20) | 0.29 | chr13 | cg20260570 | C13orf34;C13orf37 | -0.08 (-0.11, -0.05) | 0.23 |
| chr12 | cg09636302 | HAL | -0.11 (-0.15, -0.06) | 0.32 | chr18 | cg17242353 |  | 0.14 (0.08, 0.20) | 0.30 |
| chr4 | cg08611402 | ANTXR2 | 0.12 (0.07, 0.18) | 0.35 | chr4 | cg08611402 | ANTXR2 | 0.12 (0.07, 0.18) | 0.34 |
| chr12 | cg11551902 | FOXM1;C12orf32 | -0.14 (-0.20, -0.08) | 0.51 | chr20 | cg11336672 | RBL1 | 0.14 (0.08, 0.20) | 0.34 |
| chr2 | cg07419021 | TBR1 | -0.15 (-0.22, -0.09) | 0.63 | chr17 | cg04435975 | LOC404266;HOXB6 | 0.08 (0.04, 0.11) | 0.63 |
| chr4 | cg14712262 | ZFYVE28 | -0.05 (-0.08, -0.03) | 0.63 | chr5 | cg26479667 |  | -0.13 (-0.18, -0.07) | 0.63 |
| chr8 | cg24258108 | WHSC1L1 | 0.10 (0.05, 0.14) | 0.63 | chr21 | cg01233397 |  | 0.10 (0.05, 0.14) | 0.63 |
| chr20 | cg11336672 | RBL1 | 0.13 (0.07, 0.19) | 0.63 | chr1 | cg22559942 | TIMM17A | -0.05 (-0.07, -0.03) | 0.63 |
| BMI In Standard Care | | | | | BMI in Lifestyle Advice | | | | |
| **chr6** | **cg14666908** | **STK19;DOM3Z** | **0.05 (0.03, 0.06)** | **0.04** | **chr10** | **cg18646207** | **VAX1** | **0.07 (0.05, 0.10)** | **0.02** |
| **chr10** | **cg21348752** | **C10orf114;MIR1915** | **0.04 (0.02, 0.05)** | **0.04** | **chr6** | **cg14666908** | **STK19;DOM3Z** | **0.04 (0.02, 0.05)** | **0.02** |
| **chr9** | **cg01263574** | **TMEM8C** | **0.03 (0.02, 0.05)** | **0.04** | **chr9** | **cg01263574** | **TMEM8C** | **0.03 (0.02, 0.04)** | **0.02** |
| chr15 | cg07015412 |  | -0.07 (-0.09, -0.04) | 0.06 | **chr10** | **cg21348752** | **C10orf114;MIR1915** | **0.03 (0.02, 0.04)** | **0.02** |
| chr7 | cg22005393 | DNAJC2 | -0.04 (-0.06, -0.03) | 0.06 | **chr3** | **cg01919208** | **LAMB2** | **-0.06 (-0.08, -0.04)** | **0.02** |
| chr10 | cg18646207 | VAX1 | 0.08 (0.05, 0.11) | 0.07 | **chr1** | **cg26197530** |  | **-0.04 (-0.06, -0.03)** | **0.02** |
| chr3 | cg01919208 | LAMB2 | -0.06 (-0.09, -0.04) | 0.07 | **chr11** | **cg07400751** |  | **-0.05 (-0.06, -0.03)** | **0.03** |
| chr10 | cg16310045 | TCF7L2 | -0.04 (-0.06, -0.02) | 0.07 | **chr11** | **cg09585333** | **FAM89B** | **0.02 (0.02, 0.03)** | **0.03** |
| chr16 | cg06397424 | NDE1;KIAA0430 | -0.05 (-0.08, -0.03) | 0.07 | **chr2** | **cg16616765** | **HECW2** | **-0.03 (-0.05, -0.02)** | **0.03** |
| chr3 | cg24576535 |  | -0.06 (-0.08, -0.04) | 0.07 | **chr3** | **cg05654765** | **LAMB2** | **-0.06 (-0.08, -0.04)** | **0.04** |
