# Supplementary material for: Effect of an antenatal diet and lifestyle intervention and maternal BMI on cord blood DNA methylation in infants of overweight and obese women: The LIMIT Randomised Controlled Trial
Source: PLoS One. 2022 Jun 24;17(6):e0269723. doi: 10.1371/journal.pone.0269723 (PMC9231808; doi:10.1371/journal.pone.0269723)

Supplementary Material: S3 Figures

Effect of an Antenatal Diet and Lifestyle Intervention and Maternal BMI on Cord Blood DNA Methylation in Infants of Overweight and Obese Women: the LIMIT Randomised Controlled Trial

# Figure 1 (a) and (b): Results of Candidate Gene Analyses: PPARGC1A


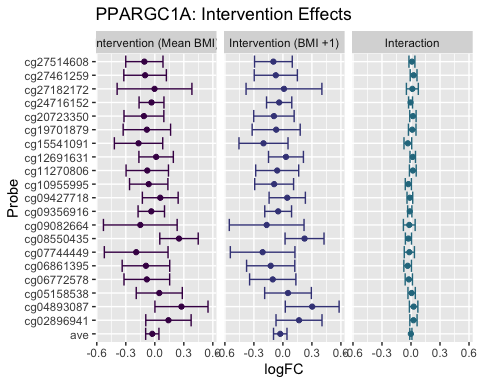

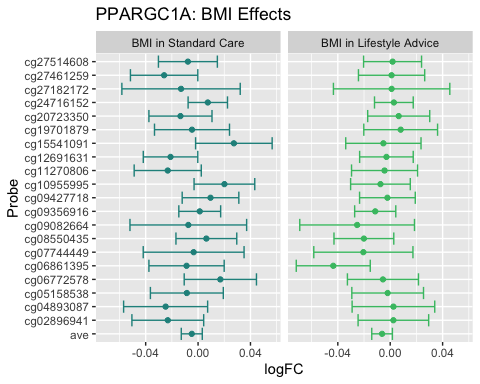


## Figure 2 (a) and (b): Results of Candidate Gene Analyses: *IGF2*


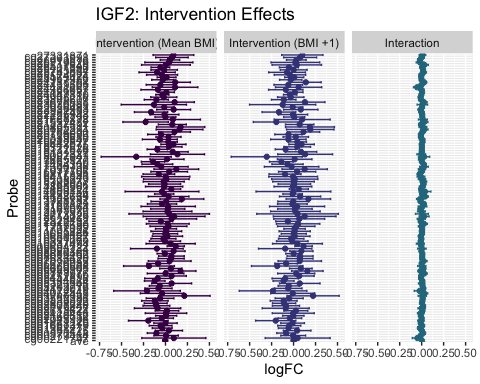

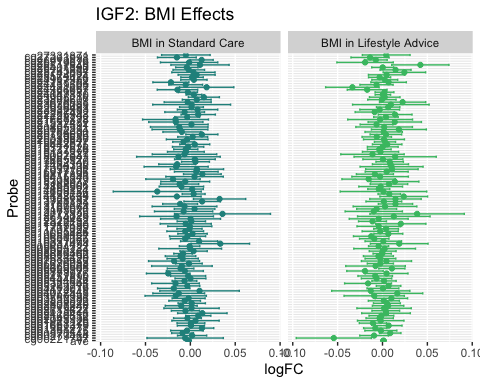


## Figure 3(a) and (b): Results of Candidate Gene Analyses: *RXRA*


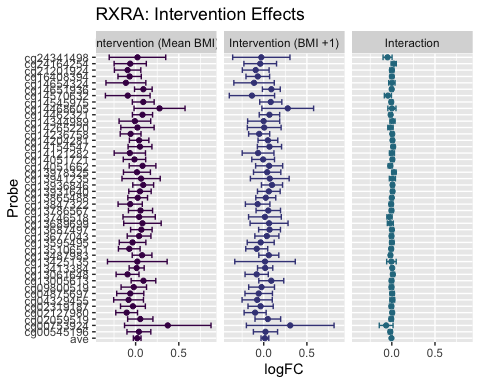

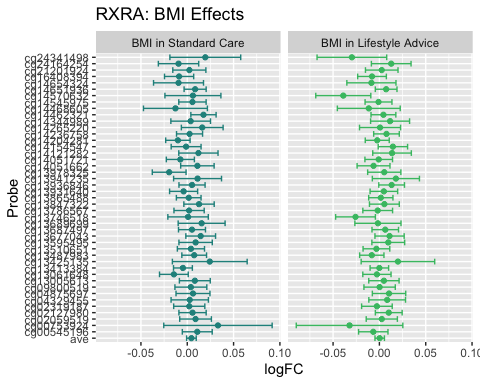


## Figure 4 (a) and (b): Results of Candidate Gene Analyses: *MEST*


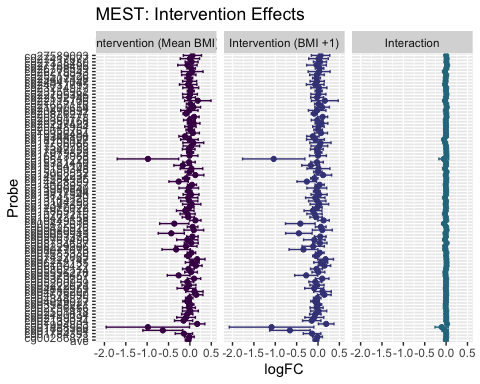

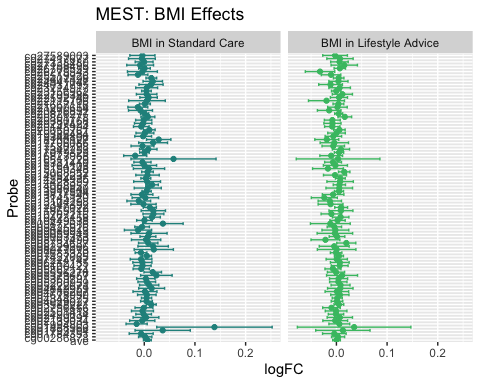

Supplement: S1 File — (DOCX) [file pone.0269723.s003.docx]
