# Supplementary material for: Effect of an antenatal diet and lifestyle intervention and maternal BMI on cord blood DNA methylation in infants of overweight and obese women: The LIMIT Randomised Controlled Trial
Source: PLoS One. 2022 Jun 24;17(6):e0269723. doi: 10.1371/journal.pone.0269723 (PMC9231808; doi:10.1371/journal.pone.0269723)
Supplement: Attachment — Submitted filename: Response to Reviewers.docx [file pone.0269723.s004.docx]

**PONE-D-21-39849**

**Effect of an antenatal diet and lifestyle intervention and maternal BMI on cord blood DNA methylation in infants of overweight and obese women: the LIMIT Randomised Controlled Trial**

**PLOS ONE**

Dear Diane and Jeunarine,

Thank you for the opportunity to revise our manuscript for publication in PLOS ONE. Point-by-point responses to reviewer and editor comments are given below. Responses to original comments appear first, followed by further editor comments and questions in the email dated Thursday 31 March 2022.

**1. Please ensure that your manuscript meets PLOS ONE's style requirements, including those for file naming. The PLOS ONE style templates can be found at**

[**https://journals.plos.org/plosone/s/file?id=wjVg/PLOSOne_formatting_sample_main_body.pdf**](https://protect-au.mimecast.com/s/cZCfCK1Dx2i8qrD3CA64Te?domain=journals.plos.org) **and**

[**https://journals.plos.org/plosone/s/file?id=ba62/PLOSOne_formatting_sample_title_authors_affiliations.pdf**](https://protect-au.mimecast.com/s/ra1VCL7Ey2SNPYkECjMNLd?domain=journals.plos.org)

We have updated the manuscript to conform to PLOS ONE style requirements according to the documents linked above.

**2. Please provide additional details regarding participant consent. In the ethics statement in the Methods and online submission information, please ensure that you have specified what type you obtained (for instance, written or verbal, and if verbal, how it was documented and witnessed). If your study included minors, state whether you obtained consent from parents or guardians. If the need for consent was waived by the ethics committee, please include this information.**

Written informed consent to participate in the LIMIT study was obtained from all participants (who were all ≥ 18 years of age). Additional written consent was obtained to collect samples of umbilical cord blood at delivery, and participants were informed that this would be used for gene expression research related to the diet and lifestyle intervention, and to weight.

This information has been added to the ethics statement, which (per (4) below) has been moved to the Methods section, lines 135-145).

**3. We note that the grant information you provided in the ‘Funding Information’ and ‘Financial Disclosure’ sections do not match. When you resubmit, please ensure that you provide the correct grant numbers for the awards you received for your study in the ‘Funding Information’ section.**

We have expanded the ‘Financial Disclosure’ statement to match the information in the original manuscript, and ensured that the grant numbers are correct. To conform with PLOS ONE style requirements, we have removed this information from the manuscript itself.

**4. Your ethics statement should only appear in the Methods section of your manuscript. If your ethics statement is written in any section besides the Methods, please move it to the Methods section and delete it from any other section. Please ensure that your ethics statement is included in your manuscript, as the ethics statement entered into the online submission form will not be published alongside your manuscript.**

The ethics statement has been moved to the methods section and deleted from ‘Declarations’ (lines 135-145).

**5. Please upload a copy of Figure 2, to which you refer in your text on page 14 and 16. If the figure is no longer to be included as part of the submission please remove all reference to it within the text.**

Apologies for the error; ‘Figure 2’ should refer to the bottom panels of Figure 1. We have amended the manuscript accordingly.

**6. Please include captions for your Supporting Information files at the end of your manuscript, and update any in-text citations to match accordingly. Please see our Supporting Information guidelines for more information:** [**http://journals.plos.org/plosone/s/supporting-information**](https://protect-au.mimecast.com/s/7OSyCMwGz2Ix562EU1g0Ui?domain=journals.plos.org)**.**

Supporting Information is now divided into three files (S1 Table, S2 Tables ad S3 Figures), described in lines 581-587 after the references, and the references to this information in the main text have been updated throughout to match the Supporting Information guidelines.

**7. Please review your reference list to ensure that it is complete and correct. If you have cited papers that have been retracted, please include the rationale for doing so in the manuscript text, or remove these references and replace them with relevant current references. Any changes to the reference list should be mentioned in the rebuttal letter that accompanies your revised manuscript. If you need to cite a retracted article, indicate the article’s retracted status in the References list and also include a citation and full reference for the retraction notice.**

We have checked the list of references to ensure correctness and completeness. No references have been retracted, and we have not made any changes to the list of references.

**5. Review Comments to the Author**

**Reviewer #1: Interesting piece of research, deepening our understanding of epigenetic mechanisms.
There is an error on Table 1 (Baseline characteristics of participants): the table reports that 85% of the participants were smokers, but the text states the contrary.**

Thank you for pointing out this error (85% of participants were *non*smokers); it has been corrected. (Line 212 Table 1).

**Reviewer #2: My major concern is that the study may not have sufficient statistical power to detect the small effect size. The power and sample size were not calculated for this secondary analysis. This raises the question whether the results are really negative or the sample size is not enough to detect it.**

Insufficient statistical power is always a potential explanation where differences are not statistically significant; since statistical significance is a function of sample size, a large enough sample would allow detection of statistically significant differences between two groups even though these may not be clinically meaningful.

Our sample size is larger than many other studies of cord blood DNAm relating to antenatal interventions or maternal overweight/obesity, including those which have found statistically significant differences. It provides 80% power to detect differences as small as 0.2 standard deviations between groups; while in the context of high-dimensional data (and resulting multiple-comparisons issues) the question of statistical power is more complicated, we believe that we were adequately powered to detect robust and clinicdally meaningful differences in DNAm.

We have added a sentence to the discussion of limitations (lines 334-337) to note that statistically significant differences may have been found with a larger sample size, but that these differences would have been of uncertain clinical significance.

**Table 1 did you compare the characteristics between the two arms? What tests were used and what are the results? All non-significant?**

We did not perform statistical tests to compare baseline characteristics at baseline; as noted in the CONSORT (Consolidated Standards of Reporting Trials) Statement Explanation and Elaboration) standards, such tests are not recommended. As the groups were randomised, it is already known that any differences between the groups are due to chance. A statistical test, which estimates the probability that differences as large as those observed would arise by chance, is therefore inappropriate.

Additionally, even outside of a randomised setting, statistical significance is not a valid indicator of the presence of confounding (nor is non-significance a valid indicator of absence of counfounding). A statistically significant difference between groups is not necessarily a confounder of the effect of interest, and a non-statistically-significant difference can still confound the effect of interest.

**Tables 2 and 3 are not clearly presented.**

We are unable to address this comment without more specific information about what the reviewer found to be unclear; we believe that this may relate to the suggestion that we present main effects rather than according to the interaction, and this is addressed below.

**Any significant interaction? There is no mention of interaction results. If there is no significant interaction, you can show main effects rather than simple effects shown in Table 2 and 3.**

The interaction between intervention and maternal BMI was not statistically significant – we have clarified this in the manuscript (lines 222-223).

However, we disagree that a non-statistically-significant interaction implies that ‘main effects’ can or should be estimated instead. Firstly, the analysis model including the interaction term was the prespecified analysis for this study, and should therefore be reported rather than the results of an analysis undertaken after viewing the results of the main analysis. Secondly, the sample size required to detect interaction effects is usually many times larger than that required to detect the individual effects, and tests of interactions are therefore usually underpowered. If the interaction term is dropped (due to non-significance in an underpowered test) and ‘main effects’ are estimated instead, there is potential for the estimates of effects of interest to be biased. As can be seen from Tables 2 and 3, the ‘top 10’ probes differ substantially for effect of intervention at different values of maternal BMI (and for effect of maternal BMI in the different intervention groups).

**What does it mean “intervention at Mean BMI”. Mean BMI of overall?
“Intervention at +5 BMI” is also confusing. Mean BMI + 5 units?**

The interaction term was between intervention (2 groups) and maternal BMI (as a continuous variable). It was therefore necessary, when presenting results, to state the value of maternal BMI at which intervention effects were estimated. ‘Intervention at Mean BMI’ is the effect of intervention estimated at the mean BMI of the cohort; ‘Intervention at +5 BMI’ is the effect of intervention estimated at mean + 5 kg/m^2^ BMI. We have added some explanation to the Table 2 notes to clarify this.

**Reviewer #3: The paper is sound, and the authors worked with sufficient data to support their conclusion.
The statistical analysis is rigorous. Different analytical models were used to justify their preposition. The availability of data upon request is documented in the paper. It is well written and in sound English. The strengths presented and well stated and strong. The weakness of the study is also well stated. The implementation of similar study among similar cohort in women with normal BMI is worth considering.**

Thank you. We do intend to investigate DNA methylation in cord blood of infants born to women of normal pre-pregnancy BMI, who participated in a later study.


**While revising your submission, please upload your figure files to the Preflight Analysis and Conversion Engine (PACE) digital diagnostic tool,** [**https://pacev2.apexcovantage.com/**](https://protect-au.mimecast.com/s/HxDZCQnME2IB6NXzcOdjO9?domain=pacev2.apexcovantage.com)**. PACE helps ensure that figures meet PLOS requirements. To use PACE, you must first register as a user. Registration is free. Then, login and navigate to the UPLOAD tab, where you will find detailed instructions on how to use the tool. If you encounter any issues or have any questions when using PACE, please email PLOS at** [**figures@plos.org**](mailto:figures@plos.org)**. Please note that Supporting Information files do not need this step.**

We have done this; the figure in the resubmission is the PACE Adjusted file.

**Responses to further comments and questions:**

**1. Please amend your Response to Reviewers letter to include a point by point response to each of the points made by the Editor and / or Reviewers. Please follow this link for more information:** [**http://blogs.PLOS.org/everyone/2011/05/10/how-to-submit-your-revised-manuscript/**](https://protect-au.mimecast.com/s/YKpUCXLWO0FMq3qwC7J-eT?domain=blogs.plos.org)

Apologies, we are somewhat confused as to how our original response to reviewers did not meet requirements (and this is not addressed in the video linked above). In this letter we have reproduced the editor and reviewer comments verbatim and given our responses.

**2. Thank you for stating the following financial disclosure:
"The LIMIT Randomised Trial was funded by an NHMRC grant (ID519240). Funding for the DNA methylation analysis was from the Commission of the European Communities, the 7th Framework Programme, contract FP7-289346-EARLY NUTRITION.

JMD was supported by NHMRC Practitioner Fellowships (ID627005 and ID1078980) and Investigator Grant (ID1196133).

BK is supported by the the European Joint Programming Initiative Project NutriPROGRAM and the German Ministry of Education and Research, Berlin (Grant Nr. 01 GI 0825). BK is the Else Kröner Seniorprofessor of Paediatrics at LMU – University of Munich, financially supported by the Else Kröner-Fresenius-Foundation, the LMU Medical Faculty and the LMU University Hospitals."

Please state what role the funders took in the study. If the funders had no role, please state: "The funders had no role in study design, data collection and analysis, decision to publish, or preparation of the manuscript."
If this statement is not correct you must amend it as needed.**
 **Please include this amended Role of Funder statement in your cover letter; we will change the online submission form on your behalf.**

Thank you, the statement regarding the role of funders is correct; funders had no role in study design, data collection and processing, the analysis plan, carrying out the analysis, decision to publish or preparation of the manuscript. We have included the statement in the cover letter as requested.

**3. Thank you for stating the following Funding Information in your manuscript:
"The LIMIT Randomised Trial was funded by an NHMRC grant (ID519240). Funding for the DNA methylation analysis was from the Commission of the European Communities, the 7th Framework Programme, contract FP7-289346-EARLY NUTRITION.

JMD was supported by NHMRC Practitioner Fellowships (ID627005 and ID1078980) and Investigator Grant (ID1196133).

BK is supported by thje the European Joint Programming Initiative Project NutriPROGRAM and the German Ministry of Education and Research, Berlin (Grant Nr. 01 GI 0825). BK is the Else Kröner Seniorprofessor of Paediatrics at LMU – University of Munich, financially supported by the Else Kröner-Fresenius-Foundation, the LMU Medical Faculty and the LMU University Hospitals"

We note that you have provided funding information that is currently declared in your Funding Statement. However, funding information should not appear in any areas of your manuscript. We will only publish funding information present in the Funding Statement section of the online submission form.

Please remove any funding-related text from the manuscript.**

Apologies, we have removed this information from the manuscript.

**4. If there are ethical or legal restrictions on sharing a de-identified minimal data set for your study, then we would require that the minimal data set be made available on request. In this case, please provide an updated data availability statement which addresses the following:

1) Explain the nature of the restrictions in detail (e.g., the minimal data set necessary to validate this study contains identifying patient-level data which cannot be suitably de-identified or aggregated.

2) State explicitly who has imposed any sharing restrictions (e.g., an ethics committee).

3) Please also provide non-author contact information (preferably institutional) for a data access committee, ethics committee, or other institutional body to which data requests may be sent. If this contact information is not institutional, please clarify the relationship of person(s) listed as data access points of contact to the data underlying your study.

4) If applicable, please provide any necessary information which interested researchers would need when requesting access to data in order to obtain the minimal data set for your study.**


The minimal dataset for this study includes participants’ personal and health information (age, BMI, parity, smoking status, quintile of socioeconomic disadvantage) along with epigenetic data. Participants consented to specific use of this data for the purposes of research related to the effect of overweight and obesity, and of an antenatal diet and lifestyle intervention, on gene expression in infants. Consent was not sought for unspecified future research (including deposition in a public repository). Under the NHMRC National Statement on Ethical Conduct in Human Research (specifically chapters 2.3 and 3.1), any use of the data not covered by the scope of the original consent requires either that consent be sought from participants, or that a waiver of consent be granted by the ethics committee. As such, it is the determination of the WCHN Human Research Ethics Committee that these data may not be made available in a public repository.

To facilitate sharing of data, an established process has been in place since the commencement of the LIMIT study. Data access requests, describing the proposed use(s) of the data, may be made by contacting the Women’s and Children’s Health Network Human Research Ethics Committee, 72 King William St., North Adelaide, South Australia, 5006 ([HealthWCHNResearch@sa.gov.au](mailto:HealthWCHNResearch@sa.gov.au)), and the LIMIT Data Access Committee (University of Adelaide, WCH Campus, 72 King William St, North Adelaide, South Australia 5006 (email ATTN: LIMIT Data Access Committee to [limit@adelaide.edu.au](mailto:limit@adelaide.edu.au)). The WCHN HREC are independent of the LIMIT study and are responsible for granting waiver of consent for the proposed research. The LIMIT data access committee will provide access to the data once this approval has been granted.
